# The Healing Effect of *Arnebia Euchroma* Ointment versus Silver Sulfadiazine on Burn Wounds in Rat

**Published:** 2015-07

**Authors:** Ebrahim Nasiri, Seyed Jalal Hosseinimehr, Mohammad Azadbakht, Jafar Akbari, Reza Enayati-Fard, Sohail Azizi, Masoud Azadbakht

**Affiliations:** 1 Traditional and Complementary Medicine Research Center, Faculty of Pharmacy, Mazandaran University of Medical Sciences, Sari, Iran;; 2 Department of Pharmaceutics, Faculty of Pharmacy, Mazandaran University of Medical Sciences, Sari, Iran;; 3 Department of Laboratory Medicine, Faculty of Allied Medical Sciences, Mazandaran University of Medical Sciences, Sari, Iran;; 4 Sana Institute, Mazandaran, Sari, Iran

**Keywords:** *Arnebia euchroma*, Burns, Silver sulfadiazine, Rat, Wound healing

## Abstract

**BACKGROUND:**

Burn is still a majordevastating condition in emergency medicine departments among both genders and all age groups in all developed and developing countries, leading to physical, psychological scars and economical burden. The present study aimed to determine the healing effect of topical treatment with *Arnebia euchroma *on second-degree burn wound in rats.

**METHODS:**

Fifty rats were divided into 4 equal groups receiving the ointment base, normal saline (NS), standard 1% silver sulfadiazine (SSD), and 5% and 10% *Arnebia euchroma *ointments (AEO). The mean of burn area, percentage of wound contraction, histopathological and bacteriological assessments in the injured area were dtermined during the study.

**RESULTS:**

Average area of wound on the 10^th^ day was 10.2±2.3, 8.4±2.6, 12.4±2.5, 5.9±2.2 and 5.7±2 cm^2^ for ointment base, NS, 1% SSD, and 5% and 10% AEO, respectively. Wound size was significantly lower in 10% AEO than 1% SSD and control groups on the 10^th^ day post-burn injury. On day 11, the percentage of wound contraction in 5% and 10% AEO was 53.9%±14.7% and 55.9±10.5% which was more than 1% SSD (15.3±10.8%). The collagen fibers were well formed and horizontally-oriented in 5% and 10% AEO groups when compared with other groups.

**CONCLUSION:**

*Arnebia euchroma* ointment was an effective treatment for healing of burn wounds in comparison with SSD and can be regarded as an alternative topical treatment for burn wounds.

## INTRODUCTION

Burn is still a major devastating condition in emergency medicine departments among both genders and all age groups in all developed and developing countries, leading to physical, psychological scars and economical burden.^1^ Thermal burn injury causes considerable incidence of disability, increase of hospitalization and rehabilitation. It is a crucial factor of morbidity and mortality, throughout the world, especially in the developing countries.^2^^,^^3^ Previous studies showed that thermal burn injury in different parts of world is high and treatment of burn wounds is a problem in public health systems.^3^^,^^4^ For survivors, the most persisting problem is scarring, so the process of wound healing and the final outcome of this process is under investigation with the hope of decreasing the problems related to scar. Burn wound healing is a complex process including inflammation, granulation, and remodeling of the tissue.^5^

There are many topical agents applied for burn wounds treatment. Silver sulfadiazine (SSD) is applied as standard topical therapy in healing of burn wounds with antibacterial activitities.^3^^,^^6^ SSD cream has several side effects such as, neutropenia, erythema multiform, methemo-globinemia, leukopenia and renal toxicity. It might also delay of the wound healing. Previous studies were reported that SSD cream should not be used for long time on extensive wounds.^3^^,^^6^ Therefore, there is a need for new agents for treatment of burn wounds in health care practice with less adverse problems and better efficacy.^7^

It is important to find more effecting drugs with shorter healing time as compare to SSD in burn injury. The use of natural products to support of the wound healing is a common practice in the world. For centuries, the medicinal plants have been extensively used in wound healing of burned injuries.^8^^-^^12^ Avicenna, the Persian scholar introduced some medicinal plants for dressing of wounds in his well-known book, Canon of Medicine, at 980-1037 AD.^13^ Today, evaluation of natural products on burns is a main part of health management and together is a capable way to offer more effective and cheaper healthcare options.^3^^,^^14^

*Arnebia euchroma* (Royle) I. Johst from the Boraginaceae family grows in different countries such as India, North Africa, Turkey, Himalaya and Iran especially in the mountain areas in north of Iran.^15^^,^^16^ *A.* roots is known as Havachoobeh in folklore and Iranian traditional Medicine (ITM) and it has been used traditionally for centuries by the Iranian people for treatment of the burn wounds as “ Marhame Havachoobeh.^17^^-^^19^ *A. euchroma* has also analgesic properties.^20^

*Arnebia euchroma* roots contains important chemical components such as naphthoquinees, shikonin, alkanin and isohexeny lnaphthazarin esters derivatives that have many pharmacological properties.^6^^,^^21^ These components have biological properties such as wound healing and antibacterial effects.^21^ This herb was evaluated to have many pharmacological properties such as anti-inflammatory,^15^^,^^22^ antimicrobial activity,^6^^,^^23^ antiamoebic, antitumor, and anticancer effects.^12^^,^^15^^,^^21^ The purpose of this study was to evaluate of the healing effect of *A. euchroma* ointment on second degree burn wounds in rats in comparison to SSD.

## MATERIALS AND METHODS

The roots of *A. euchroma* were prepared from a local herbal market in Sari in north of Iran during October 2013. It was confirmed by botanists Dr. Masoud and Dr. Mohammad Azadbakht at the Mazandaran University of Medical Science, Iran (Herbarium number: 1001). The roots of the *A. euchroma* were washed and dried in a hot air oven at 40°C for 8 hours. 

The dried materials were powdered in a grinder. Then samples were sieved and levigated with glycerine. Finally, liquid paraffin and eucerin were added and mixed. *A. euchroma* ointment (AEO) was made from *Arnebia euchroma* (10 g), glycerin (25 g), eucerin (60 g) and liquid paraffin (5 g). This formulation with the weight ratio of AEO (1:2.5:0.5: 6) was prepared as described before.^24^^,^^25^ Levigated AEO was mixed with liquid paraffin, eucerin, propyl and methyl paraben, then the composition was mixed and homogenized at 500 rpm for 10 min. This ointment containing 5 and 10% AEO were prepared identically. All formulations were filled in tubes and stored in 4, 25, and 40^0^C for two weeks and the stability of ointments were evaluated. 

The experimental protocol was approved by Ethical and Research Committee of Mazandaran University of Medical Sciences (No=118-92, 3). All animals were obtained from this university. Male Wistar rats (n=50) weighing 160-200 g with average age of 10 weeks old were used and housed under standard condition at room temperature with a 12-h light/dark cycle. Animals were allowed free access to laboratory food and water *ad libitum*. The rats were anesthetized by intraperitoneal injection of 50 mg/kg sodium thiopental. 

The dorsal portion of the body was shaved using an electrical clipper and 70% alcohol was used to disinfect the dorsal area. Deep anesthetized rats were kept in prone position. A deep second degree burn wound was caused by a hot plate (diameter: 5 cm × 2.5 cm) warmed 5 min within boiling water and put for 10 s on the skin with an equal weight and pressure as described before.^7^

All rats were immediately resuscitated with injection of 5 ml normal saline.^26^ The burned animals were randomly divided into five groups of ten rats. Group 1 was control that was only washed with normal saline (NS) during dressing without any topical treatment; group 2 was treated with the ointment base without any effective agent; group 3 was treated with 1% SSD (Behvarzan Pharmaceutical Company, Iran). Groups 4 and 5 were treated with 5 and 10% AEO, and then sterile plain gauze was covered and fixed on wound for 24 hours. 

All wounds were washed with normal saline, and wound in each group was applied with cream and dressed. Wounds were dressed once per day. Rats were washed with normal saline before dressing in all groups. In order to quantify the rate of wound healing, the size of lesions was determined on 1, 3, 7, 10, 15, 20, 25, and 30 days after burn injury. The wound area was displayed as cm^2^. The area of wounds at each day was determined by a formula, where represents the area (cm^2^) by length, and latitude rectangular. Percentage of wound contraction at each time point was derived by the following formula: percent wound contraction=(initial wound area-current wound area)/initial wound area×100%. 

Also, the sum of the scales in each part of histopathological evaluation was divided to four groups. We evaluated extent of granulation tissue, the new dermis, collagen organization and re-epithelization, according to Table 1 as a histological study.^27^^-^^29^ Histopathological conditions of the complete burn wound healing and re-epithelialization was evaluated on 7 and 21 days after burn injury. Burned skin tissue samples were taken for histological studies with a small excision containing part of the wound area. Tissue samples were fixed in 10% formalin solution. Paraffin embedded tissue section of 4 μm were prepared and tissue processing stained with hematoxylin and eosin. Light microscopy was used to assessing pathological changes. 

**Table 1:** Scales for the burn wound healing accordind to histopathological components

**Sum of components variables**	**Difinition of Scales**
Extent of granulation tissue (7 parameters) [(-3)-18]	(-3-0)=not healing (1-4)=low (5-12)=moderate healing (13-18)=good wound healing.
Re-epithelization (6 components) [0-18]	0=not healing (1-6)=low (7-12)=moderate healing (13-18)=good wound healing.
New dermis (5 parameters) [0-15]	0=not healing (1-5)=low (6-10)=moderate healing (11-15)=good wound healing.
Sum of three components [0-57]	[(-3)-0]=not healing [1-18)=low (19-37)=moderate healing (38-57)=good wound healing

These tissue sections were assessed by a blinded pathologist. The average of ten microscopic fields was recorded by him for each specimen. Histopathological criterias were defined according to a modified scoring system for surgical wound healing taken from previous studies according to the sum of the scales for wound healing divided to three groups.^27^^-^^29^ Extent of granulation tissue was scored based on seven parameters on 7^th ^day includin (i) Monocytic macrophage (0-3), (ii) Neovascularization (0-3), (iii) Fibroblastic proliferation (0-3), (iv) Degree of granulation tissue (0-3), (v) Matrix (mucopolisacarid deposition) (0-3), (vi) Degree of inflamation (0-3), and (vii) Extend of bacterial colonization (-3 to 0).

The sum of scales related to granulation state was a range from -3 to 18. The 18 value was highest degree of granulation tissue formation on the 7^th^ day after burn injury while [-3-0)]=absent granulation, [1-4]=low, [5-12]=moderate, and [13-18] was determined as good extent of granulation tissue. The re-epithelization was monitored by evaluation of six components as following on the 7^th^ day including (i) Epidermal thickness extent with (0-3), (ii) Thickness of granular cell layer (0-3), (iii) Maturation and organization of squamous cells (0-3), (iv) Extent of keratin layer (0-3), (v) Orthokeratin (0-3) and (vi) Parakerotosis (0-3). In full thickness, thin with completely well organized and showed with score of 3(+++). The sum of scales related to re-epithelization was at range of 0 to 18. The 18 value was the highest degree of re-epithelization on the 7^th^ day after burn injury including 0=no epitilialization, [1-6)]= low, [7-13]=moderate, and [14-18] was determined as good re-epithelization.^27^^-^^29^

The histopathological examination of the complete wound healing or the new dermis was scored based on five components as follows: (i) Degree of scar formation (0-3), (ii) Mmatrix of collagenization organization (0-3), (iii) Extend of hair folliculs (0-3), (iv) Extent of lymphatic ducts (0-3), and (v) Degree of innervation (0-3). The sum of score was at a range between 0 to 15. The best condition for new dermis was scored 15 on 21^st^ day after burn injury. The sum of scales for wound healing were devided to four groups as follows: 0=no healing, (1-5)=low, (6-10)=moderate healing, and (11-15)=good healing.^27-^^29^ The sum of scales for each part of histopathological evaluation was divided to three groups (extent of granulation, new dermis, and re-epithelization) as shown in Table 1.

Main parameters of new dermis, and collagen organization were defined at four levels, and each level was scored and defined as follows: (-) or 0=lagging down disorganized and poorly oriented collagen fiber none or absent, (+) or 1=10-20% collagen fibers are horizontally oriented, (++) or 2=30-40% collagen fibers are horizontally oriented, and (+++) or 3=well formed and horizontally oriented collagen fiber. Complete healing was evaluated by degree of scar formation, collagenization organization, hair follicles and degree of innervations after three weeks of burn induction. A score was defined for all parameters evaluated:including 0= absent, 1=mild presence, 2=moderate presence and 3=strong presence.^30^

Swabs were taken from the burn wound during dressing change on 4^th^ and 8^th^ days. The collected swabs were immediately transferred to the laboratory for microbial test. In the quantitative count study, 0.5 ml of normal saline was added to each of the samples. Each sample dilution was spread onto blood and Maccankey agar, and the plates were incubated at 37°C for 24 hours. Diagnostic test for the colonies was applied with neopucine.

Wound sites were assessed daily. Macroscopic visual evaluation was measured by direct observation of wound during dressing each day. Inflammatous tissue was defined to have edema, secretion, redness, dark secretion or pus, pain and wound bleeding during dressing washing. At each follow-up visit, the wound area was noted as being absent for any secretion, and have red, edematous, dirty and light or dark secretions.

Statistical analysis was carried out using the SPSS and Excel software. One-way analysis of variance (ANOVA) was used to compare variables in all groups. Post Hoc multiple comparisons Tukey test was used to compare the means and also, Kruskal Wallis H test for qualitative variables between groups (SPSS, version 15.0 for windows, Chicago, IL, USA). The differences were considered significant at *p*<0.05.

## RESULTS

The effect of burn injury on losing weight of rat was shown in Table 2. No significant differences were noticed between AEO and control groups for weight loss after burn injury. Skin wounds were measured on days 1, 3, 7, 10, 15, 20, 25 and 30 post-burn injury. The average area of wound on the 7^th^ day was 11.2±2.4, 9.9±1.7, 13.1±1.6, 10.3±2.5 and 9±2 cm2 in ointment base, NS, 1% SSD, 5 and 10% AEO, respectively (*p*<0.002). The wound sizes were not significantly different between groups on the 1^st^ and 3^rd days after^ burn injury. 

**Table 2:** Mean weights (gram) of the rats after burn injury

**Group** *	**1** ^st^ ** day**	**3** ^rd^ ** day**	**7** ^th^ ** day**	**15** ^th^ ** day**	**25** ^th^ ** day**	**30** ^th^ ** day**
BO	183±16	191±18	196±21	209±24	219±31	227±27
N/S	183±13	191±13	197±12	209±12	227±15	239±11
SSD	184±12	190±11	197±11	209±11	228±12	241±9
5% A	182±18	192±21	198±20	210±21	225±22	237±14
10% A	182±17	190±18	197±17	209±15	226±9	239±21
*P value*	0.999	1	0.998	1	0.874	0.515

* Ointment base (BO), normal saline (N/S), 1% silver sulfadiazine (1% SSD) and 5 and 10% *Arnebia euchroma* ointment (5% A, 10% A).

On the 7^th ^day of the post-burn injury, wound area was significantly different between groups following multiple comparison by Tukey post Hoc test showing that NS, 5 and 10% AEO exhibited a lower wound size as compared to 1% SSD at the 7^th^ day after burn injury, respectively (*p*<0.03, *p*<0.04 and *p*<0.001) (Table 3). There was not any evidence of noticeable skin irritation such as erythema, edema and inflammation during the study period.

**Table 3:** Mean wound area (cm^2^) of the animals treated with various topical ointments post-burn injury

**Group** *	**1** ^st^ ** day**	**3** ^rd^ ** day**	**7** ^th^ ** day**	**10** ^th^ ** day**	**15** ^th^ ** day**	**20** ^th^ ** day**	**25** ^th^ ** day**	**30** ^th^ ** day**
BO	13.1±2.8	14.2±2	11.2±2.4	10.2±2.3	5.1±1.5	1.8±0.6	1±0.6	0.4±0.3
N/S	11.9±2.5	12.5±2.2	9.9±1.7	8.4±2.6	6.9±2.2	3.9±1.5	0.7±0.4	0.08±0.1
SSD	13.5±2.2	13.9±2.6	13.1±1.6	12.4±2.5	10.5±1.5	5.5±1.5	3.1±1.3	1.9±0.8
5% A	13±3.8	14.6±2.6	10.3±2.5	5.9±2.2	2.4±0.6	0.4±0.3	0	0
10% A	12.8±2.2	15.8±2.9	9±2	5.7±2	2.1±0.3	0.4±0.3	0	0
*P value*	0.795	0.089	0.002	0.001	0.001	0.001	0.001	0.001

* Ointment base (BO), normal saline (N/S), 1% silver sulfadiazine (1% SSD) and 5 and 10% *Arnebia euchroma* ointment (5% A, 10% A).

**Fig. 1:**
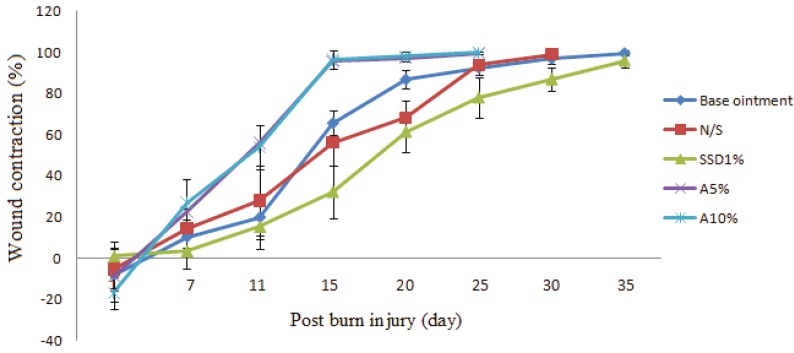
Comparison of the percentage of wound contraction between *Arnebia euchroma *and other groups. 5 and 10% *Arnebia euchroma* ointment showed a faster wound contraction

The wound area was not significantly difference between control groups (1% SSD, ointment base and NS) on the 10^th^ day of post-burn injury. The difference was significant between 5 and 10% AEO and SSD. No significant difference was noticed regarding the wound size between 5 and 10% AEO treated groups on 10^th^ and 15^th^ days after burn injury. The percentage of wound contraction in different groups was shown in Table 4 showing a significant increase in the 5 and 10% AEO groups when compared to the control group. Animals receiving 1% SSD ointment had a longer healing time in comparison to rats in the AEO treatment groups. These findings were shown in Figure 1. It is clear that wound contraction started from day 5 in all groups, which was faster in the 5 and 10% AEO groups as compared to the control group. On day 7, animals treated with 10% AEO exhibited significant increase in the percentage of wound contraction as compared to other experimental groups (29.7% in 10% AEO as compared to 3.4% in SSD, 14.4% in ointment base and 16.2% in NS groups).

**Table 4:** Comparison of the percentage of wound contraction between *Arnebia euchroma *and other groups

**Group** *	**3** ^rd^ ** day** **(%)**	**7** ^th^ ** day (%)**	**11** ^th^ ** day (%)**	**15** ^th^ ** day (%)**	**20** ^th^ ** day (%)**	**25** ^th^ ** day (%)**	**30** ^th^ ** day (%)**	**35** ^th^ ** day (%)**
BO	-8.6±12.7	10±8.7	19.8±10.6	65.5±6	86.7±4.4	92.3±3	96.8±2.3	99.4±1.2
N/S	-6.4±10.6	14.6±9.7	27.9±16.8	56±11.2	68.2±8.1	93.8±2.4	98.9±1.5	98.6±1.8
1% SSD	-2.7±7	3.5±8.4	15.3±10.8	32.2±12.6	61.3±9.6	77.9±9.8	86.8±5.5	95.8±3.2
5% A	-8.6±7.6	22.5±11.5	53.9±14.7	95.5±4.3	97.1±2.7	99.6±.6	100	100
10% A	16.8%±6.3	26.9±10.6	55.9±10.5	96.3±3.6	97.9±2.3	100	100	100

* Ointment base (BO), normal saline (N/S), 1% silver sulfadiazine (1% SSD) and 5 and 10% *Arnebia euchroma* ointment (5% A, 10% A).

**Fig. 2:**
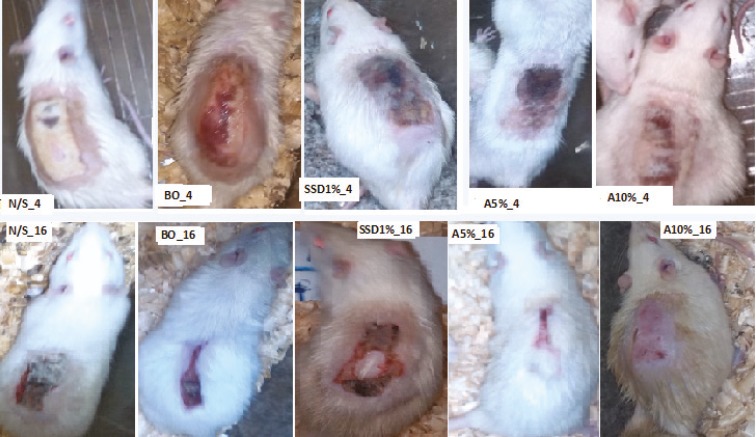
Burn wound healing patterns in Normal saline (N/S), ointment Base (BO), 1% silver sulfadiazine (1% SSD) and 5 and 10% *Arnebia euchroma* ointment (5% A, 10% A) in rats after 4 and 16 days. Average wound size was smaller and the healing rate was faster in 5% A and 10% *Arnebia euchroma* ointment than other groups; *p*<0.001.

On day 15 of post-burn injury, 5% and 10% AEO showed about 95% wound healing, whereas it was 32.2%, 56% and 65.5% for rats treated with %1 SSD, NS and ointment base, respectively. On day 25, No scar was seen in animals treated with 10% AEO, while this improvement was observed for 1% SSD group after day 35. A completed wound healing was noticed in animals treated with 5% and 10% AEO on 25^th^ day, while this time period was 35^th^ day for 1% SSD and control groups. It is clear that wound care in the rats treated with AEO was at least 10 days shorter than 1% SSD and the control groups (Table 4 and Figure 1). The condition of wound healing on the 4^th^ and 16^th^ days after burns was illustrated in Figure 2.

**Fig. 3:**
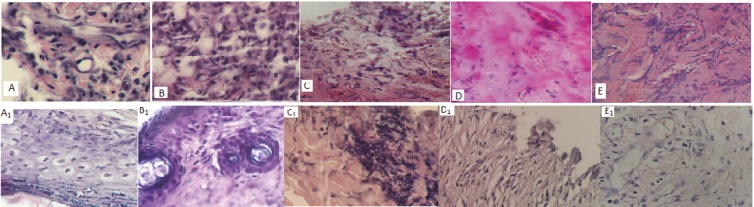
Comparison of the histopathology of samples from second degree burn wounds on 7^th^ and 21^st^ days after treatment with ointment base (BO), normal saline (N/S), silver sulfadiazine (1% SSD), and 5% *Arnebia euchroma* (5% A) and 10% *Arnebia euchroma* (10% A). Macrophage infiltration, neovascularization and fibroblastic proliferation were more in 10% A, and 5% A, compared with 1% SSD, BO and N/S groups on the 7^th^ day. Also, thickness of granular cell layer and epidermal thickness extent were prominent in *Arnebia euchroma* groups in comparison with other groups on 7^th^ and 21^st^ days. A=*5*% *Arnebia euchroma*_7^th^ day, A1=*5*% *Arnebia euchroma*_21^st^ day, B=10% *Arnebia euchroma*_7^th^ day, B1=10% *Arnebia euchroma_*21^st^ day, C=1% Silver sulfadiazine_7^th^ day, C_1_=1% Silver sulfadiazine_21^st^ day, D=Ointment base_7^th^ day, D_1_=Ointment base_21^st^ day, E=Normal saline_7^th^ day, E_1_=Normal saline_21^st^ day.

The extent of granulation tissue formation, matrix organization, re-epithelialization and formation of a new dermis were determined. On day 7, samples revealed an increase in macrophage and cellular infiltration in animals treated with 5 and 10% AEO in comparison to SSD and NS groups. The extent of maturation, tissue organization and re-epithelialization was more in groups treated with 5 and 19% AEO when compared with SSD and ointment base groups (Figure 3). 

At the 7^th^ day of the experiment, histopathological evaluation showed the highest formation of granulation tissue for *A. euchroma* and the lowest extent for NS group (Table 5). The re-epithelialization parameter for 5% AEO treated group was significantly more than 1% SSD and controls groups (*p*<005) (Table 6). The collagen organization in 10% AEO group was better than 1% SSD and control groups on 21^st^ day. The score of collagen organization in 10% AEO, SSD, 5% AEO, ointment base and NS groups was 3, 2, 2, 1 and 2, respectively (Table 7). 

**Table 5:** Histopathology of granulation tissue formation among different groups on the 7^th^ day after burn injury

**Components/Groups** *	**BO**	**1% SSD**	**N/S**	**5% A**	**10% A**
Macrophage histocytic infiltration	3	2	2	3	3
Neovascularization	2	2	2	3	3
Fibroblastic proliferation	2	2	2	3	3
Matrix mucopolisacharide deposition	1	2	2	2	2
Degree of inflammation	2	3	1	3	3
Extent of bacterial colonization	-1	-2	-1	-1	-1
Degree of granulation tissue formation	2	2	1	3	3
Total	11	11	9	16	16

* Ointment base (BO), normal saline (N/S), 1% silver sulfadiazine (1% SSD) and 5 and 10% *Arnebia euchroma* ointment (5% A, 10% A).

**Table 6:** Comparison regarding re-epithelialization between groups on the 7^th^ day post-burn injury

**Group** * **/Components**	**Epidermal thickness (0-3)**	**Thickness of granular cell layer**	**Maturation organization of squamous cells**	**Extent of keratin layer**	**Orthokeratin**	**Parakeratosis**	**Total**
BO	1	1	1	1	1	0	5
N/S	10	0	0	0	0	0	0
1% SSD	1	1	1	1	1	1	6
5% A	3	3	2	2	2	0	12
10% A	3	3	3	3	3	0	15
*P value*							0.001

* Ointment base (BO), normal saline (N/S), 1% silver sulfadiazine (1% SSD) and 5 and 10% *Arnebia euchroma* ointment (5% A, 10% A).

**Table 7:** Comparison of new dermis formation between groups on the 21^st^ day post-burn injury time

**Groups** *	**Degree of scar formation**	**Collagenization/ organization**	**Extent of hair follicles **	**Extent of lymphatic ducts**	**Degree of innervations**	**Total**
5% A	2	2	2	1	0	7
10% A	3	3	3	2	1	12
1% SSD	2	2	1	0	0	5
Base Ointment	2	1	1	1	1	6
N/S	2	2	1	0	0	5

* Ointment base (BO), normal saline (N/S), 1% silver sulfadiazine (1% SSD) and 5 and 10% *Arnebia euchroma* ointment (5% A, 10% A).

Histopathological findings showed a complete wound healing and new dermis formation in 10% AEO, whereas 5% AEO and 1% SSD and ointment base groups showed a moderate wound healing score. The assessment of granulation tissue formstion demonstrated that SSD and ointment base groups when compared with AEO groups (5 and 10%) did not have any significant differences, whereas NS group did have a significant difference with AEO treated groups (*p*<0.38). Formation of new dermis in AEO groups was better than SSD and control groups (*p*<0.017). The sum of three main histopathology components of wound healing were shown in Table 7.

According to histopathological findings (Table 8), 5 and 10% AEO treated groups had a good healing, 1% SSD and ointment base groups had moderate healing and NS group had a low wound healing score. No edema, inflammation or discharges were seen in rats treated with AEO and SSD during the study. The period of re-epithelialization time among the study groups were different. The re-epithelialization time for 10% AEO was 20.3±4 days and for 5% AEO, NS, ointment base and 1% SSD was 20.8±3.6, 29.5±3.1, 33±3.6 and 34.7±3 days, respectively (*p*<0.001). 

**Table 8:** The sum of the Scales of three histopathological components for burn wound healing

**Group**	**Re-epithelialization** **[0-15]**	**Extent of granulation tissue [(-3)-18]**	**New dermis formation [0-15]**	**Sum of scales** **[(-3)-58]**
BO	5	12	6	23
N/S	1	9	5	15
1% SSD	6	11	5	22
5% A	12	16	7	35
10% A	15	16	12	43
*P value*				*p*<001

* Ointment base (BO), normal saline (N/S), 1% silver sulfadiazine (1% SSD) and 5 and 10% *Arnebia euchroma* ointment (5% A, 10% A).table

A complete wound healing was observed in animals treated with 5 and 10% AEO on 20^th^ day, while this time was 34.7 days for 1% SSD and almost similar for the other control groups. It is clear that wound care in the rats treated with AEO was 9-14 days shorter than the control groups. Visual observations of the burn wounds in the NS and ointment base groups showed a moderate secretion, redness and edema in the wound area, whereas wounds treated with AEO and 1% SSD showed a negligible redness and edema without any secretion. 

The laboratory assessments indicated no evidence of bacteria. In the 4^th^ and 8^th^ days, a little secretion appeared in wounds in some rats treated with ointment base, NS, SSD and 5% AEO. These samples were cultured on blood agar and were positive for growth of Gram positive cocci while were sensitive to neopucine that was considered as an epidermal normal flora.

## DISCUSSION

The purpose of this study was to evaluate the effect of AEO on healing of burn wounds in experimental rats. Results showed a significant improvement in wound healing of rats treated by topical 5 and 10% AEO during the experimental period, as compared with 1% SSD and other control groups. Topical AEO were much more effective in wound healing than 1% SSD, ointment base and NS groups. Wound size was remarkable less in 5 and 10% AEO groups as compared with control and 1% SSD groups after 7 days. AEO was did not show any toxicity and side effects.^18^ The root of *A.* *euchroma* used to prepare AEO has been used in Iranian traditional medicine in different parts of Iran and also in other countries for treatment of wound healing. 

Many chemical components have been identified in A. *euchroma *roots and leaves from Boraginaceae family.^15^^,^^26^ Alkannins and Shikonins existed in the external layer of the roots of the *A. euchroma.* Pharmaceutical formulations with wound healing properties based on alkannins and shikonins have been in the market for many years. Previous studies revealed that *A. euchroma *was rich in naphthoquinones, hydroxynaphthoquinone, phenolic acids, alkaloids, shikonins and Alkannins.^21^^,^^27^ It was shown that shikonins derivatives and alkannins from *A. euchroma *and Boraginaceae family had anti-inflammatory and wound healing properties.^15^^,^^19^

In addition, antibacterial,^28^ anti-inflammatory,^22^ antioxidant, antiviral, antifungal, antitumor, anticancer,^21^^,^^26^^,^^27^ and analgesia effects,^17^^,^^26^^,^^27^ of *A. euchroma* have been reported beofore. In the present work, our findings denoted to the efficiency of *A. euchroma *root which was superior to 1% SSD in burn wound healing. 

The anti-inflammatory, antibacterial and antioxidant properties of *A. euchroma* would be a potential to improve wound healing. Our finding showed that 5% and 10% AEO could effectively prevent secondary complications of burn such as swelling, erythema and secretion or wound infection that may to due to antibacterial properties of this ointment. Improved rate of wound contraction and reduction was observed in animal treated with *A. euchroma *roots. This result is consistent with other reports of wound healing of the most other species of Boraginaceae family, raising the possibility that this family has a wound healing potential.^16^^,^^22^

The wound healing rate of *A. euchroma *was shorter than that of the standard 1% SSD ointment. A delay in burn wound healing was observed in the ointment base, NS and 1% SSD groups when compared with 10% AEO. In the AEO group in comparison to the 1% SSD treated group, less complications were visible explaining the delay in wound healing in control groups as compared to ARO group. Although previous study showed that 1% SSD ointment was a standard ointment in burn wounds, but it was associated with longer hospitalization.^3^^,^^28^^,^^29^

In histopathological assessment, re-epithelialization was significantly more in animals treated 10% AEO. Re-epithelialization was dependent on the thickness of granular cell layer, epidermal thickness extent, maturation and organization of squamous cells and migration of epithelial cells that all these components showed an increasing trend. Previous studies reported the anti-inflammatory effect of some Boraginaceae species and development of collagen fibers and epithelium regeneration that were accelerated and also the epithelium thickness improved.^16^^,^^28^^,^^29^

In this study, the wound repair process in AEO group was better than 1% SSD and other control groups regarding the degree of inflammation, mucopolysacharid deposition, fibroblastic proliferation, macrophage infiltration, granulation tissue formation and neovascularization. No pathogenic organisem was noticed when AEO was administered. 

*A. euchroma* treatment measure lead to an increase in neovascularization, short period of epithelialization and absence of any infection. Re-epithelialization significantly increased in the 10% AE group in comparison to other groups. Collagen plays an essentials role in wound healing and as a principal component of connective tissue provides a structural framework for the tissue regeneration. Collagen is produced by fibroblasts affecting the healing process during wound healing.^30^ The effects of AEO on wound healing is through changes in cell regeneration and collagen synthesis and density of blood vessels.^25^^,^^26^ Wound healing process is complex and occurs during coagulation, inflammation, debridement and re-epithelialization phases and is associated with proliferation, migration and integration of squamous epithelial cells of the epidermis. In the end stage of the healing process, collagen deposition and remodeling occurs in the dermis.^31^ The granulation tissue formation and re-epithelialization on the 7^th^ day post-burn injury and formation of the new dermal tissue on the 21^st^ day was better in AEO group when compared with other groups. In most cases, the AEO in high concentration showed better healing effects than the other groups. One possible assumption for this could be due to important phytochemical constituents of *A. euchroma* for wound healing such as flavonoids, alkannin derivatives, according to the ITM program, Marhame Havachoobeh being used traditionally for centuries by Iranian people for treatment of the burn wounds. Formulation of the Marhame Havachoobeh was simple. It was prepared based on sesame oil and wax. Also, the roots of Boraginaceae family plants soaked in butter were used in treatment of local wounds.^17^^,^^18^^,^^24^ Also, the roots of Boraginaceae family plants soaked in butter are used in treatment of local wound. *A. euchroma* ointment was prepared on eucerin. This simple levigated ointment may be better released from constituents in the Arnebia roots.^16^^,^^32^ Results of this study showed that preparation of *A. euchroma* roots is simple and with low cost and effective in burn wound healing in comparison to silver sulfadiazine. Therefore, topical administration of *A. euchroma* ointment can be an alternative in healing of burn wounds.

## CONFLICT OF INTEREST

The authors declare no conflict of interest.

## Note

Please cite this paper as:

Nasiri E, Hosseinimehr SJ, Azadbakht M, Akbari J, Enayati-Fard R, Azizi S, Azadbakht M. The Healing Effect of *Arnebia Euchroma* Ointment versus Silver Sulfadiazine on Burn Wounds in Rat. World J Plast Surg 2015;4(2):134-144.
